# Alpha-1 Antitrypsin Deficiency-Associated panniculitis: A survey of lived experience

**DOI:** 10.1371/journal.pone.0326686

**Published:** 2025-06-26

**Authors:** Jaiden Townsend, Reem Alluhibi, Nicky Lynch, Richard Woolf, Aileen Marshall, David A. Lomas, John R. Hurst

**Affiliations:** 1 UCL Respiratory, University College London, London, United Kingdom; 2 Department of Respiratory Care, King Saud bin Abdulaziz University for Health Sciences, Jeddah, Saudi Arabia; 3 King Abdullah International Medical Research Centre, Jeddah, Saudi Arabia; 4 London, United Kingdom; 5 St John’s Institute of Dermatology, Guy’s and St Thomas’ NHS Foundation Trust, London, United Kingdom; 6 Hepatology, Royal Free London NHS Foundation Trust, London, United Kingdom; Medizinische Fakultat der RWTH Aachen, GERMANY

## Abstract

**Introduction:**

Alpha-1 Antitrypsin Deficiency (AATD)-associated panniculitis is a rare inflammatory condition characterized by painful subcutaneous plaques or nodules, often accompanied by ulceration or oily discharge. Despite its clinical and emotional burden, limited data exist on the lived experiences of individuals with this condition.

**Method:**

An international online survey was conducted between April and July 2024, targeting individuals with AATD-associated panniculitis. The survey, co-designed with an affected individual and multi-disciplinary specialists, included 32 questions on demographics, diagnostic journey, life impact, and treatments. Responses were analysed using descriptive statistics and thematic analysis.

**Results:**

41 responses were included in the analysis (68% female; mean age 52.3 years). Participants reported lesions at diverse sites, affecting the lower and upper limbs, followed by the trunk, buttocks, and genitalia. 41.5% experienced both ulceration and oily discharge. 61% of participants reported being misdiagnosed which negatively affected their mental health. More than half of respondents ‘strongly agreed’ or ‘agreed’ that living with alpha-1 panniculitis had made them anxious. Access to specialist care was a major concern, with 69% finding it difficult to obtain specialist advice. Treatments varied, and augmentation therapy was identified as the subjectively the most effective. Open-ended responses revealed gaps in healthcare professionals’ awareness and highlighted the need for better mental health support and specialist access.

**Conclusion:**

AATD-associated panniculitis significantly impacts physical, emotional, and social well-being. Addressing gaps in diagnosis and treatment, increasing healthcare providers awareness, and adopting multidisciplinary approaches are essential to improve individuals’ outcomes and quality of life.

## Introduction

Alpha-1 antitrypsin deficiency (AATD) is a genetic disorder most commonly caused by the homozygous ZZ genotype affecting one in 2000−5000 individuals [1]. It is characterised by deficiency or abnormality of the alpha-1 antitrypsin (AAT) protein, produced by the liver and plays a crucial role in protecting the lungs from damage caused by neutrophil enzymes [1]. The typical pulmonary manifestation of AATD is early onset panlobular emphysema [2], In individuals with the ZZ type of AATD, The abnormal accumulation of misfolded AAT within the liver may present as neonatal hepatitis, and cirrhosis, and hepatocellular carcinoma [1,3].

Panniculitis is a heterogenous group of inflammatory conditions defined by inflammation of the subcutaneous adipose tissue [4]. The inflammation typically results in painful nodules or plaques beneath the skin, often accompanied by redness, swelling, and warmth [5]. People with lived experience emphasise the severity of the pain and how difficult this can be to manage. Different autoinflammatory conditions are associated with panniculitis and the presentation is often determined in part by the underlying cause [6]. The presentation of AATD as a cause of panniculitis is rare as both conditions are uncommon, with an estimated prevalence of panniculitis of 1 per 1000 individuals with AATD [7]. Panniculitis associated with AATD presents as recurrent painful plaques and nodules, often following local trauma [8,9]. Lesions are thought to typically affect the proximal limbs and/or buttocks, and there can be overlying skin breakdown/ulceration with suppurative/oily discharge [10]. Deep skin biopsy, including subcutaneous fat, can be helpful in diagnosis as there are certain typical histological features of AATD-associated panniculitis such as spread of neutrophils into the reticular dermis and septa of the subcutis and skin Z polymers [11,12]. The diagnosis of AATD panniculitis is usually established through a combination of clinical findings, serum AAT concentration, and/or AATD phenotyping or genotyping [13]. Despite its clinical relevance, AATD-associated panniculitis remains poorly understood, with limited literature documenting its presentation and impact. A systematic review in 2022 identified only 117 cases, highlighting the scarcity of data, particularly regarding long term disease course and outcomes [13]. Furthermore, no studies have explored the lived experiences of individuals with AATD-associated panniculitis, leaving a significant gap in understanding. This study sought to investigate the lived experiences of individuals with AATD-associated panniculitis.

## Method

### Materials and methods

We co-designed and conducted a cross sectional international online survey for people living with AATD-associated panniculitis. The survey was live between 26^th^ April and 15^th^ July 2024 using a web-based survey tool. The study was approved by UCL Ethics committee and conducted according to Good Clinical Practice. All participants provided informed consent before completing the survey. Participants were recruited online through social media platforms including Facebook and X. Dissemination of the survey was assisted by alpha-1 patient organizations (Alpha-1 Foundation in the US and Alpha-1 UK).

The eligibility criteria were individuals who had self-reported AATD-associated panniculitis. The survey was designed and revised by a team of AATD specialists including respiratory physicians, a hepatologist, a dermatologist and a person with lived experience. The survey was piloted with six individuals including two with AATD-associated panniculitis, and changes were made accordingly. The final survey contained 32 questions divided into three sections: (1) demographics, (2) closed questions about the journey to diagnosis, effect of the condition on daily life, and access to treatment graded using a Likert scale. Responses were graded “strongly agree”, “agree”, “neither agree nor disagree”, “disagree”, “strongly disagree” or “not applicable”; (3) five open-ended questions covering other key aspects of lived experience.

### Data analysis

SPSS V.28 was used for data analysis, which was predominantly descriptive, reporting proportions. A thematic analysis approach was applied to analyse free text. Following the framework of Braun and Clarke (2006) [14], we identified patterns and themes within the data, with some themes divided into sub-themes. An inductive approach to thematic analysis was employed to identify recurring responses and insights.

## Results

### Participants

**Fig 1:**
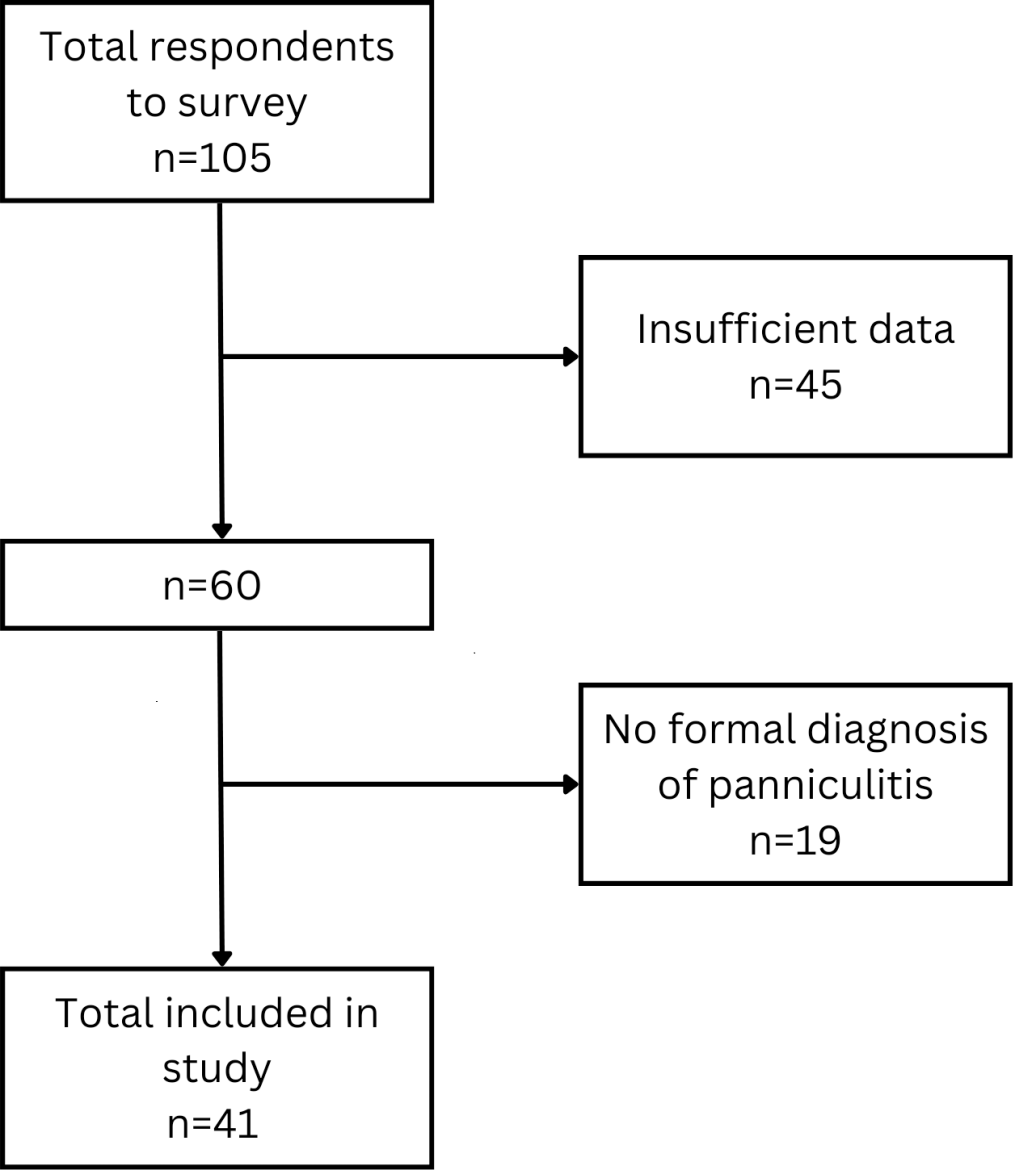
Responses to the survey.

A total of 105 individuals responded to the survey. After eliminating responses from people who did not meet the eligibility criteria of both AATD and panniculitis, 41 participants were included in the final analysis (Fig 1). The mean age of participants was 52.3 ± 14.8 years and 68% (n = 28) were female, other characteristics are reported in Table 1. The majority of responses were from Europe, and mainly from the UK 54% (n = 22), with 17% (n = 7) from the USA. In 75% (n = 31) of respondents, AATD was diagnosed before the panniculitis. The mean age of AATD diagnosis was 37.1 ± 16.1 years, while the mean age of panniculitis diagnosis was 41.3 ± 14.1 years.

**Table 1:** Baseline characteristics of the survey participants.

Characteristics	n (%)
Respondents	41
Age (year)	
Mean	52.3
SD	14.8
Gender	
Male	11 (26.8)
Female	28 (68.3)
Other	2 (4.9)
Alpha-1 Phenotype	
ZZ	19 (46.3)
SZ	4 (9.8)
SS	1 (2.4
MZ	9 (22.0)
MS	1 (2.4)
Other	5 (12.0)
Unknown	2 (4.9)
Lungs affected	
Yes	27 (65.9)
No	7 (17.1)
Unknown	7 (17.1)
Liver affected	
Yes	10 (24.4)
No	19 (46.3)
Unknown	12 (29.3)

### Characteristics of panniculitis lesions

**Fig 2:**
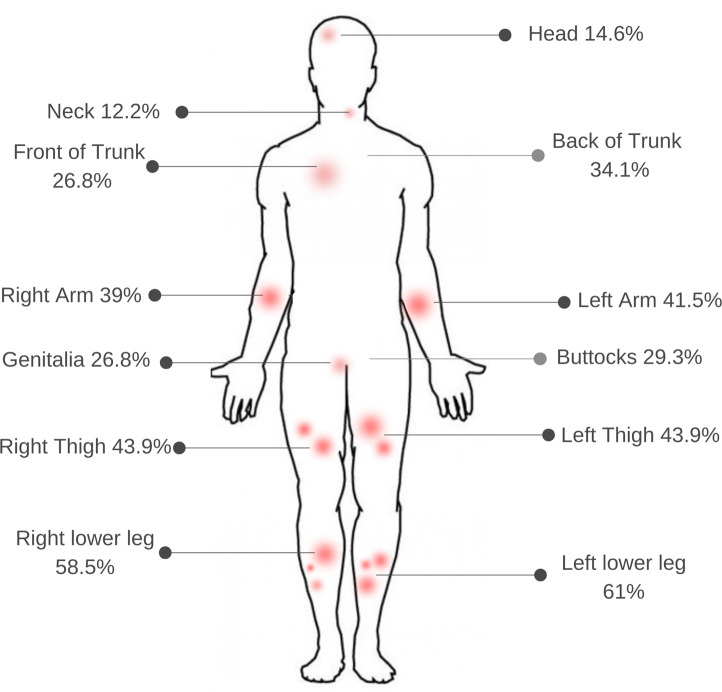
Reported frequency of lesions locations among survey participants.

The distribution of panniculitis lesions among respondents revealed multiple patterns. The thighs and lower legs were most commonly affected; 18 respondents (44%) reported lesions on the thighs and 25 respondents (60%) reported lesions on the lower legs. Six of these individuals (14%) had lesions that exclusively affected the thighs and lower legs. Lesions on the back of the trunk and arms were also common. The frequency across all locations is illustrated in (Fig 2). Seven respondents (17%) had lesions exclusively affecting sites other than the legs.

When asked to describe the lesions, 17 participants (41.5%) reported both ulceration and oily discharge, four participants (9.8%) experienced ulceration alone, two participants (4.9%) experienced oily discharge alone, and 18 participants (43.9%) reported neither of these characteristics.

### Journey to diagnosis and impact on daily life

**Fig 3:**
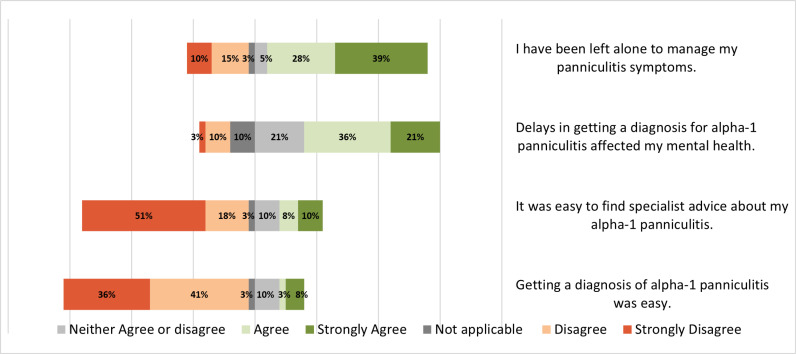
Journey to diagnosis.

Thirty-nine percent and 28% of respondents ‘strongly agreed’ and ‘agreed’ respectively that they had been left alone to manage their panniculitis symptoms. Furthermore, 57% ‘strongly agreed’ or ‘agreed’ that delays in getting a diagnosis for their alpha-1 panniculitis had negatively impacted their mental health. 51% of respondents ‘strongly disagreed’ to the statement that it was easy to find specialist advice about alpha-1 panniculitis and 77% ‘strongly disagreed’ or ‘disagreed’ with the statement that getting a diagnosis of alpha-1 panniculitis was easy. Other responses relating to the diagnostic journey are illustrated in (Fig 3).

**Fig 4:**
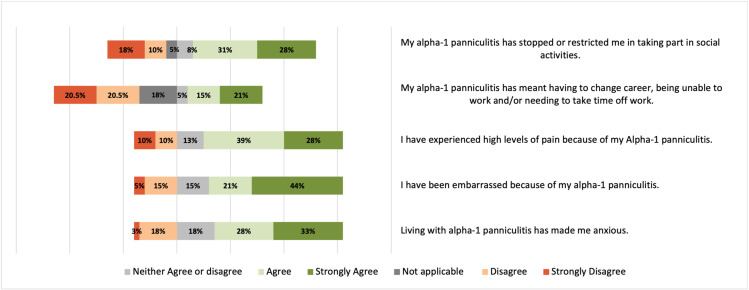
Effect on daily living.

67% ‘strongly agreed’ or ‘agreed’ that they experienced high levels of pain because of their condition, 46% of participants ‘strongly agreed’ that they have been embarrassed because of their condition and 61% ‘strongly agreed’ or ‘agreed’ that living with AATD-associated panniculitis had made them anxious. 59% of respondents ‘strongly agreed’ or ‘agreed’ that the condition had restricted or prevented them from participating in social activities and 36% of individuals ‘strongly agreed’ or ‘agreed’ that AATD-associated panniculitis had affected their professional lives, resulting in career changes, an inability to work or the need to take time off from their jobs. Other responses related to the effect on daily life are illustrated in (Fig 4).

The results of the survey point to barriers in proper diagnosis and treatment as 61% of respondents reported misdiagnosis and 49% reported not receiving specific treatment. Of the people who had received treatment (total n = 17), 24% had received alpha-1 augmentation therapy, 17% dapsone, steroids and augmentation, 12% dapsone alone and 12% received steroids alone. AAT augmentation therapy was reported by 7/13 individuals (54%) as being the most effective treatment that they had received.

### Thematic analysis

When discussing the challenges of communicating with healthcare professionals (HCPs) about their diagnosis, many participants described a lack of knowledge by HCPs. One participant remarked, “[doctors have] *no knowledge or understanding of the link between AATD and panniculitis*.” The location of lesions emerged as a significant barrier with two participants citing it as a particularly sensitive topic. Their responses included references to “*genital lesions*” and “*boils and lesions on the bottom and testicles*.”

Participants gave a variety of responses when discussing the support that they found helpful since diagnosis. The analysis is presented in Table 2.

**Table 2:** The themes and example quotations from participants’ responses about support.

Themes	Sub-themes	Examples of participant responses
Positive	Websites	*“I have read quite a bit myself and obtained information through various websites and alpha1 associations”* *“This group” [sic. Facebook page]* *“Alpha 1 support groups”*
	Doctors	*“Dermatologist, familiar, with alpha-1 and panniculitis”* *“Finding a specialist”* *“The doctors”* *“Support from the doctors who have cared for me and continue to care for me”* *“Support from doctors and hospital employees”*
	Medical centers	*“Rare disease center Birmingham* [UK]*”*
	Family	*“My family who were with me during the hardest moments”*
	Friends	*“Some friends who were with me during the hardest moments”*
Negative	No support	*“None”* *“No support”* *“Not received any”* *“Haven’t had any”*

When asked about the support they would like to see for managing AATD-associated panniculitis, many participants expressed concern about the widespread lack of knowledge and awareness. Participants emphasised the need for “*more widely available information*,” “*greater knowledge and skills in this area*” and “*better education for doctors*.” Another prominent theme was the need for specialist care, with one participant stating the importance of “*more specialist availability and increased awareness of Alpha-1*”. The effectiveness of current treatment options also emerged as a key concern. Some participants discussed augmentation therapy, with one noting that “*augmentation therapy isn’t enough*”, while another expressed a desire for timely access to this treatment, stating, “*I would like to see patients get augmentation therapy, so it clears up quickly before they end up in the hospital*”. Mental health support was also highlighted as a crucial aspect of treatment with one participant calling for “*immediate mental health support and counselling*”.

Participants highlighted a range of gaps in healthcare they experienced, which have been analysed and summarized in Table 3.

**Table 3:** Themes about gaps in the current healthcare system concerning the diagnosis.

Themes	Sub-themes	Examples of participant responses
Lack of knowledge	GPs	*“Professionals do not have enough knowledge especially GPs”* *“I’m never taken seriously. The GP has never heard of it so just shrugs it off”*
	Medical professionals	*“…I have found there is a significant lack of knowledge about Alpha-One generally, let alone the aspect of panniculitis. Whilst coughing my heart out, I was told by an ED doctor that my cough was not related to Alpha-One as this only affected the liver.”* *“Lack of awareness of condition & correct way to treat it (and how not to treat it), more information to educate GP’s & other* *consultants whether in A&E or dermatology etc.”*
	Available information	*“Lack of information. I never knew that panniculitis can occur with alpha-1”* *“Understanding of both conditions by one person”*
No gaps	None	*“My experience is that there are none. When I was diagnosed almost 30 years ago, I was asked to participate in a medical conference on rare skin diseases. I never got to know anything after this participation.”* *“Not in my experience”*

Participants were asked to reflect on the advice that they would give to someone who suspects that they have AATD-associated panniculitis. A prominent theme was the importance of persistent engagement with healthcare providers. For instance, participants advised, “*keep persisting with questions through your GP. Do not be fobbed off with creams and words*” and “*find a specialist as soon as possible to begin treatment*”. The need for thorough testing was another recurring theme with suggestions such as “*insist on DNA tests and diagnostic tests*” “*get genetic tests as soon as possible*,” and “*insist on Alpha-1 testing*”. Self-education also emerged as crucial with participants recommending “*research as much as possible before going to your doctor*” and “*get as much information from doctors, ask questions, and request testing*”. Lastly, the theme of perseverance was highlighted with advice such as “*Don’t give up until you get the right diagnosis*”, “*Keep going back until someone listens to you*” and “*Never give up*”.

## Discussion

This survey describes the lived experience and self-reported characteristics of AATD-associated panniculitis, and highlights the burden and challenges faced by individuals with this uncommon condition.

Most participants were AAT Z homozygotes, however MZ, MS, SS and SZ heterozygote phenotypes were also reported. This is consistent with the limited existing literature. In a case series of ten individuals with AATD-associated panniculitis, five were Z homozygotes with a recent systematic review identifying fewer than ten MS heterozygotes affected by AATD-associated panniculitis [10,13].

The most common sites of panniculitis lesions were the lower legs and thighs which is also in line with previous case series [15,16]. However, many participants reported lesions on the trunk, arms, neck and head. Fewer than half the participants experienced both ulceration and oily discharge: Geraminejad and colleagues suggested that ulceration and oily discharge are distinctive features of AATD panniculitis compared with other forms of panniculitis [17].

Despite many participants being diagnosed with AATD prior to developing panniculitis, the rate of misdiagnosis was high. While lungs and liver involvement are well-recognized features of AATD, the presence of these manifestations did not necessarily lead to a timely or accurate diagnosis of panniculitis. This highlights a gap in clinical practice: even individuals with a previous diagnosis of AATD may struggle to receive appropriate evaluation for associated conditions such as panniculitis.

Living with AATD-associated panniculitis involves enduring a significant burden of symptoms that affect both physical and emotional well-being [18,19]. The very high levels of pain reported by participants underscore the physical burden of the condition, that can be particularly disruptive in performing daily tasks. The emotional burden of AATD-associated panniculitis was also high with many participants reporting anxiety and noted that delays in diagnosis negatively impacted on their mental health. Living with a rare condition such as AATD-associated panniculitis often involves navigating uncertainty, compounded by the frustration of frequent misdiagnosis and difficulty of accessing specialist advice and treatments [19].

This emotional toll is further amplified by a perceived lack of support with many participants feeling left to manage their symptoms alone. These findings reflect broader challenges faced by individuals with rare diseases. In addition, the impact of panniculitis extended beyond physical and emotional challenges, affecting socioeconomic outcomes such as career changes and the need for time off work, reducing quality of life.

The findings from our study have several implications for clinical practice. First, the high rates of misdiagnosis, delays to diagnosis and delays in receiving treatment highlight the need for increased education and awareness among healthcare providers. Many respondents expressed frustration over the difficulty in finding specialist advice. Earlier diagnosis and intervention may significantly improve individuals’ outcomes and reduce the mental health impact. In addition, a multidisciplinary approach that includes dermatologists, pulmonologists and mental health professionals may be necessary to best address the needs of these individuals. Lastly, more advocacy and support networks are needed to ensure that individuals with this rare condition have access to the resources and the care that they require.

Although the results from this survey are informative and the first to our knowledge to specifically reflect individuals’ experience living with AATD-associated panniculitis, it is important to note that our survey has limitations. Our data are self-reported and so there are assumptions regarding the accuracy of the respondents’ diagnoses, and it is subject to recall bias. In addition, recruitment through patients support groups may have enrolled more highly engaged, motivated care seekers who might have faced more challenging care journeys.

In conclusion, we provide the first in-depth understanding of the lived experience of individuals with AATD-associated panniculitis, highlighting the significant physical, mental, and social challenges they face. We also describe the location and characteristics of the lesions. The findings emphasize the need for greater awareness among healthcare providers, better signposting to expert advice be that medical or charity-based, and improved access to effective treatments. Addressing these gaps will be critical in improving the quality of life for individuals living with this rare condition.

## Supporting information

S1 Survey instrument. (DOCX)
