# Supplementary material for: Alpha-1 Antitrypsin Deficiency-Associated panniculitis: A survey of lived experience
Source: PLoS One. 2025 Jun 26;20(6):e0326686. doi: 10.1371/journal.pone.0326686 (PMC12200858; doi:10.1371/journal.pone.0326686)
Supplement: S1 — (DOCX) [file pone.0326686.s001.docx]

Demographic / Introduction

Section 1

Do you consent to taking part in this survey?

- Yes
- No

1. What is your gender?

- Male
- Female
- Other / Prefer not to say.

2. What is your age?

3. What type of alpha-1 deficiency do you have?

- MS
- MZ
- SS
- SZ
- ZZ
- Other …………….

o I don’t know.

4. Which country do you live in? ……….

5. How old were you when you were first diagnosed with alpha-1 antitrypsin deficiency?

6. How old were you when you were first diagnosed with panniculitis?

7. Which was diagnosed first?

- Alpha-1 antitrypsin deficiency
- Panniculitis

8. Are your lungs affected by alpha-1?

- Yes
- No
- I Don’t Know

9. Is your liver affected by alpha-1?

- Yes
- No
- I Don’t Know

10. Where has the panniculitis been located on your body (choose all that apply)?

- Head
- Neck
- Front of Trunk
- Back of Trunk
- Right Arm
- Left Arm
- Buttocks
- Genitalia
- Right Thigh
- Left Thigh
- Right lower leg
- Left lower leg

11. Have you ever had ulceration or oily discharge from your panniculitis lesions?

- Ulceration
- Oily discharge
- Both
- Neither

Section 2

Please select the option which most accurately reflects your agreement/disagreement with the following statements.

- - Strongly agree.
  - Agree.
  - Neither agree nor disagree.
  - Disagree.
  - Strongly disagree.
  - Not applicable.

1. Getting a diagnosis of alpha-1 panniculitis was easy.

2. Delays in getting a diagnosis for alpha-1 panniculitis affected my mental health.

3. It was easy to find specialist advice about my alpha-1 panniculitis.

4. I have been left alone to manage my panniculitis symptoms.

5. Living with alpha-1 panniculitis has made me anxious.

6. I have been embarrassed because of my alpha-1 panniculitis.

7. I have experienced high levels of pain because of my Alpha-1 panniculitis.

8. My alpha-1 panniculitis has meant having to change career, being unable to work and/or needing to take time off work.

9. My alpha-1 panniculitis has stopped or restricted me in taking part in social activities.

10. I have not been able to access treatment for alpha-1 panniculitis because alpha-1 replacement or ‘augmentation’ treatment is not available where I live.

11. My healthcare is provided through health-insurance, and I have not been able to access alpha-1 replacement or ‘augmentation’ treatment for alpha-1 panniculitis because my insurance will not cover it.

12. I have not been able to access alpha-1 replacement or ‘augmentation’ treatment for alpha-1 panniculitis because I cannot afford to pay for it.

Section 3

Please do not include any personal data in the open text boxes below.

1. Were you initially misdiagnosed?

- - Yes
  - No
  - If yes, what was alpha-1 panniculitis diagnosed as?

2. Were there aspects of your condition that were particularly difficult to talk to health care professionals about?

- - Yes
  - No
  - If yes, what were these aspects?

3. Have you had any specific treatments for alpha-1 panniculitis?

- - Yes
  - No
  - If yes, Which treatments have you received?

4. Which treatments worked best?

5. How effective was the treatment that worked best?

- - Not effective at all
  - Slightly effective
  - Somewhat effective
  - Very effective
  - Extremely effective

6. What kind of support have you found most helpful?

7. What kind of support would you like to see?

8. In your experience, what gaps exist in the current healthcare system concerning the diagnosis and management of alpha-1 panniculitis?

9. Looking back, what advice would you give to someone who suspects they may have alpha-1 panniculitis, based on your own diagnostic journey and experiences with healthcare?
